# First Complete Genome Sequence of a Feline Alphacoronavirus 1 Strain from Brazil

**DOI:** 10.1128/MRA.01535-18

**Published:** 2019-03-07

**Authors:** Bruno de Cássio Veloso de Barros, Ceyla Maria Oeiras de Castro, Diego Pereira, Laila Graziela Ribeiro, José Wandilson Barboza Duarte Júnior, Samir Mansour Moraes Casseb, Gustavo Moraes Holanda, Ana Cecília Ribeiro Cruz, Edivaldo Costa Sousa Júnior, Joana D’Arc Pereira Mascarenhas

**Affiliations:** a Section of Virology, Evandro Chagas Institute, Ministry of Health, Ananindeua, Pará, Brazil; b Section of Arbovirology and Hemorrhagic Fevers, Evandro Chagas Institute, Ministry of Health, Ananindeua, Pará, Brazil; KU Leuven

## Abstract

We identified a strain of Alphacoronavirus 1, FCoV-SB22, from a pool of fecal samples from domestic cats from a rural settlement in the municipality of Santa Bárbara, Pará, Brazil. The nucleotide identity with feline coronavirus was 91.5%. The present study reports the first complete genome sequence of a feline coronavirus from Brazil.

## Abstract (precis)

We identified a strain of Alphacoronavirus 1, FCoV-SB22, from a pool of fecal samples from domestic cats from a rural settlement in the municipality of Santa Bárbara, Pará, Brazil. The nucleotide identity with feline coronavirus was 91.5%.

## ANNOUNCEMENT

Feline coronaviruses (FCoV) are common pathogens in domestic and wild cats in several regions of the world and may cause lethal infections, such as feline infectious peritonitis (FIP) (1). These viruses are classified in the order *Nidovirales*, family *Coronaviridae*, and genus Alphacoronavirus and are subdivided into two serotypes, FCoV 1 and FCoV 2. The FCoV virion is pleomorphic and enveloped and contains a single-stranded positive-sense RNA genome (27 to 32 kb). The genome of the species Alphacoronavirus 1 has 11 open reading frames (ORFs) (2, 3). In domestic cats, FCoV 1 is predominant, leading to subclinical infections (4). In December 2016, 5 fecal samples from nondiarrheic domestic felines aged 5 months to 6 years were collected from a rural community located in the municipality of Santa Bárbara in the state of Pará, Brazil. These samples were pooled and processed for Illumina sequencing. A cDNA library was prepared using a Vilo superscript reverse transcriptase (RT) and sequenced on an Illumina MiniSeq platform using the methodology described in the Nextera XT DNA library preparation kit (5), using paired-end reads with 150 bp.

The genome was assembled using a hybrid methodology of *de novo* assembly and reference mapping with the Minia (kmer-size = 32, abundance-min = 3, abundance-max = 500, nb-cores = 8) (6) and Geneious (with default parameters) version 8.1.9 (7) programs, respectively.

Taxonomic annotation was performed using the Kraken software (with default parameters) (8). The DNA sequencing produced 8,137,466 reads. Using a *de novo* assembly methodology, 39,267 contigs were generated, 25 of which were related to the family *Coronaviridae*, 107 that were for uncultured cross-assembly phage (crAssphage), 2 that were for RD114 retrovirus, 1 that was for feline picornavirus, 4,987 that were for bacteria, and 34,140 that were undetermined by Kraken 1.0. The *Coronaviridae* contigs were characterized as Alphacoronavirus 1 FCoV and after reference mapping generated a single unitig formed by 1,597 reads, which was related to feline coronavirus strain UG-FH8, isolated from Denmark (GenBank accession no. KX722529). The new FCoV-SB22 strain showed a genome of 29,137 bp, exhibiting 91.5% nucleotide identity with the strain used for reference mapping. The average read coverage was 8×, and the GC content was 38.7%. We report here the almost complete genome sequence of Alphacoronavirus 1 obtained from nondiarrheic domestic felines that inhabited rural environments in Brazil. The role of this pathogen in feline infection remains poorly understood, and more studies are needed for a better understanding of its role. Therefore, the continuous surveillance of this agent and the improvement of diagnostic methods and their implementation are needed, in addition to strategies to prevent and control infection.

### Data availability.

The complete genome sequence reported here was deposited in GenBank under the accession no. MH817484 and in the SRA under accession no. SRR8352624.
